# Combined Assay of rDNA and SatIII Copy Numbers as an Individual Profile of Stress Resistance, Longevity, Fertility and Disease Predisposition

**DOI:** 10.3390/jpm12101752

**Published:** 2022-10-21

**Authors:** Lev N. Porokhovnik

**Affiliations:** Laboratory of Molecular Biology, Research Centre for Medical Genetics, 1 Moskvorechye, 115522 Moscow, Russia; med-gen@mail.ru

**Keywords:** SatIII, satellite repeat, ribosomal repeat, rDNA, CNV, stress response, copy number

## Abstract

The ribosomal DNA and pericentromeric satellite repeats are two important types of moderately repeated sequences existing in the human genome. They are functionally involved in the universal stress response. There is accumulating evidence that the copy number variation (CNV) of the repeat units is a novel factor modulating the stress response and, thus, has phenotypic manifestations. The ribosomal repeat copy number plays a role in stress resistance, lifespan, in vitro fertilization chances, disease progression and aging, while the dynamics of the satellite copy number are a sort of indicator of the current stress state. Here, we review some facts showing that a combined assay of rDNA and SatII/III abundance can provide valuable individual data (“stress profile”) indicating not only the inherited adaptive reserve but also the stress duration and acute or chronic character of the stress. Thus, the repeat count could have applications in personalized medicine in the future.

## 1. Introduction

A repetitive nature is a fundamental principle of the organization of the human genome. Copy number variation (CNV) is an important source of natural genetic diversity. In the most recent two decades, studies have shown a variety of phenotypic manifestations of the individual copy number of various genes or sequences in the human genome. The scope of the phenotypic manifestations widely varies from adaptive traits to lethality during embryonal development. Associations have been reported between some CNV polymorphisms and the progression of severe diseases, including autism, schizophrenia, Crohn’s disease, rheumatoid arthritis, type 1 diabetes, adiposity and numerous impairments of development [1]. This review leaves aside the unique genes and only relates to the functional and prognostic role of the CNVs of two key types of human moderate repeats: ribosomal and satellite DNA.

Ribosomal repeats, also called ribosomal DNA, are genes located in human acrocentric chromosomes 13, 14, 15, 21 and 22 and coding for the 45S-pre-rRNA. In addition to their coding function, they are key players in supporting global genome stability in eukaryotic cells [2]. In lower eukaryotes and Insecta, somatic alterations (reduction or augmentation) in the ribosomal gene copy count are possible, resulting in genome instability [3] that provokes, in turn, aging, cancer and various diseases. In higher organisms, including humans, the copy number of ribosomal repeats seems to be much more stable in various cells over their lifetime. The scheme of the ribosomal DNA organization is highly conservative throughout various eukaryotic taxa [4].

Ribosome biogenesis governs the human cell’s ability to proliferate and carry out its functions normally. In the dividing cell, the polymerase I-based rRNA synthesis is most active; therefore, rRNA transcripts account for 35–60% of the total RNA amount in the cell [5]. Hence, the polymerase I transcription rate is closely associated with cell growth and proliferative activity, depending greatly on various factors, such as oxidative stress, aging, DNA damage, alimentary restrictions, etc. [6,7,8]. Dysregulation in ribosome biogenesis caused by the impaired transcription of ribosomal genes results in the progression of a number of diseases [9]. Recently, the ribosome biogenesis rate was proposed as a very sensitive sensor of cellular stress [10]. The abundance of human ribosomal DNA has the same effect, modulating ribosome content. It is a stable inherited quantitative trait with a variety of phenotypic manifestations.

The pericentric satellite III (SatIII or Sat3) and II tandem repeats are the other kinds of moderate repeats involved in the stress reaction. Historically deemed “constitutive heterochromatin”, these repetitive sequences were recently found to be transcribed by polymerase II under stress conditions, and the transcripts were shown to play an essential role in the universal stress response (reviewed in [11]).

The stress response requires the temporal and reversible silencing of the major mass of genes. The cell’s metabolism “keeps its head down” during stress in order to save energy and not waste resources for the synthesis of macromolecules, which are highly likely to be damaged under stress-inducing conditions. The non-canonical transcription of satellite repeats is now hypothesized to be a key event in stress-linked transcription arrest. Stress-induced satellite III transcription recruits polymerase II, the histone acetyltransferase CREBBP (also termed CBP) and some splicing factors to form special structures termed nuclear stress bodies, which serve as specific “traps”. As a result, the cellular pool of these molecules is exhausted to stop genome expression [12,13,14]. Below, we will try to show that the number of SatII/III copies in an individual genome can be a novel factor modulating the efficiency of the stress reaction and an indicator of stress intensity and duration.

This review is an attempt to prove that a combined assay of an abundance of two important kinds of moderate repeats occurring in the human genome, ribosomal and satellite, can provide valuable data, not only predetermining the high or low effectiveness of the individual stress response and thereby modulating the individual stress-resistance, expected lifetime, fertility and predisposition to pathology but also indicating the current state of the stress, its duration and acute or chronic character of the stress. Thus, the repeat copy data can eventually provide an individual profile of adaptive reserve, which could find application in personalized medicine.

## 2. The Importance of Ribosomal Repeat Abundance

The functional consequences of human ribosomal gene CNVs are various and multifaced. Modulating the rate of protein synthesis, the rDNA copy number is a quantitative trait linked to diverse, sometimes unexpected processes and phenotypic manifestations. Indeed, this “housekeeper” rules the whole cell’s “kingdom” through its “backstairs influence”.

At the dawn of life, the ribosomal gene CNV orchestrates the child’s development rate. The daily weight gain during the first months of life shows a statistically reliable positive correlation (r = 0.47, *p* < 0.05) with the abundance of ribosomal repeats in the infant’s genome [15]. In another study on healthy newborns (n = 99), the copy number of ribosomal genes was found to correlate with a series of parameters describing infant development, such as height, weight, head and chest circumference, age of first tooth and number of teeth by the age of six (r = 0.60), nine (r = 0.64) and 12 months (r = 0.66) [16]. Interestingly, birth weight demonstrated no link to the number of ribosomal repeats in the infant’s genome. Apparently, this parameter is generally determined by the mother’s genome.

At the end of life, the individual copy number of ribosomal genes for 45S rRNA also matters. A study was performed on a large group of 651 subjects aged 17 to 91 years, who were divided into two subgroups—an “elderly” group (E-group, N = 126) of individuals over 72 y.o. and a “non-elderly” group (NE-group, N = 525)—and the study showed that both groups had the same mean rDNA copy numbers, but the variation was narrower in the E-group than in the NE-group. Notably, the E-group genomes contained almost no hypermethylated rDNA copies, contrary to the NE-group. Earlier, human fibroblasts were found to lose hypermethylated copies of rDNA during replicative senescence in vitro. The authors hypothesized two processes that follow aging: first, subjects with pronouncedly low or high rDNA content have shortened life expectancy and, thus, are underrepresented in the elderly group, and second, the human genome loses hypermethylated rDNA copies with aging [17]. By all appearances, the first process is prevalent because such stabilizing selection does not shift the mean. If the second tendency (elimination of hypermethylated rDNA copies) prevailed, the mean rDNA copy number (CN) would decrease with aging. The finding that the average population rDNA copy number remains constant was corroborated by the findings of a qPCR-based study on young (median age 20) and old (median age 79) subjects [18].

As an alternative to the shortened lifespan required to explain constant means with lower variations in rDNA copy numbers during human aging, one could imagine that increased DNA damage during aging might stimulate mitotic recombination such that extreme copy number differences would be reduced without invoking differences in survival. However, otherwise, computer simulations have shown that unequal recombination results in increasing variations in rDNA copy numbers [19].

Similar facts suggesting a reduced lifespan in the carriers of deviant (both low and high) rDNA abundance were discovered in research comparing newborn (n = 52) and adolescent/adult (10 to 40 years old, n = 48) patients with Down syndrome, which can be regarded as a model of accelerated aging. The comparison results revealed a significant narrowing of the interval of ribosomal gene CNVs with age, while the mean remained the same. Similar findings were obtained from a sample of patients with numerical abnormalities of the gonosomes (n = 32, of which 14 individuals had Turner’s syndrome and 18 had Klinefelter’s syndrome). All the patients constituting this sample lay within the range of the medium (“adaptive”) part of the distribution of the trait. The sample showed no difference in the mean compared to that of the control group (*p* = 0.24), but rather a significant decrease in variance (1.09) compared with the healthy controls (2.56) (F = 2.34, *p* = 0.0056). These data suggest the prenatal selection of carriers of the numerical abnormalities of sex chromosomes, similar to postnatal mortality in Down syndrome patients, leading to the enhanced elimination of carriers with low and high amounts of ribosomal genes in the genome [20].

A thought-provoking ribosomal gene CNV dataset was obtained on studies of pregnant women with normal and complicated pregnancies and women who had undergone, successfully or not, one or more rounds of the in vitro fertilization (IVF) procedure. Pregnancy is a stressful load on women; therefore, all the stress-resistance factors are relevant, including individual rDNA abundance. Studies have shown that the genomes of more than half of women with complicated pregnancies harbor more or less rDNA copies than any control woman with normal pregnancies, suggesting that successful gestation requires balanced ribosome biogenesis. Woman with low and very high rDNA copy numbers belong to the risk group and need more careful attendance during pregnancy. Women with three or more failed IVF attempts had significantly fewer rDNA copies than women who were able to get pregnant after the first or second attempt. This suggests that the rDNA copy number is a factor modulating IVF success chances. The risk of IVF failure is high in female subjects carrying less than 330 rDNA copies in the genome [21].

Multiple studies of patients with diverse diseases revealed associations with a predisposition to and the severity of some pathologies with a biased low or high abundance of ribosomal DNA.

The phlegmon is a spreading diffuse inflammatory process that comes with the formation of purulent exudate. A study conducted on a sample of n = 46 patients showed that more severe purulent inflammation was associated with lower copy numbers of ribosomal genes in a patient’s genome. The patients were divided into two groups: Group 1 (n = 26), with a satisfactory general state when admitted, one anatomic area involved in the pathologic process and no complications in the follow-up period, and Group 2 (n = 20), consisting of patients with a moderately grave or severe general state when admitted, two or more anatomic areas involved in the pathologic process and complications during the follow-up period. In Group 2, the mean rDNA copy number was found to be significantly (*p* < 0.05) lower than in Group 1 [22].

Intriguingly, the individual number of copies of the “typical housekeeping gene” existing in each living cell with a nucleus seems to be able to modulate the complex processes of higher nervous activity.

One of the most well-known and, at the same time, so-far-unexplained facts is the increased mean copy numbers of ribosomal repeats in the samples of schizophrenia patients compared with healthy controls: The median rDNA copy number was 542 in a group of 179 (108 male/71 female) subjects with schizophrenia versus a median of 384 in a reference group consisting of 122 (60 male/62 female) controls (*p* = 10^−25^) [23]. The underpinning causes have yet to be elucidated.

It is noteworthy that epidemiological studies have revealed reduced comorbidity in schizophrenia and rheumatoid arthritis, a systemic autoimmune disease of the connective tissue, with small joints being predominantly affected [24,25,26,27]. The reason remains, so far, unexplained. In a group of rheumatoid arthritis patients, copy numbers of transcriptionally active rRNA genes appeared to be significantly smaller than in the controls. The copy number of the active ribosomal gene fraction in ~90% of the patients was less than the mean value in the control group [28,29].

The facts of opposite rRNA gene dosages in these two diseases might provide an opportunity for a novel interpretation of the well-known decreased rates of schizophrenia and rheumatoid arthritis comorbidity.

Reduced ribosomal activity has been suggested to be a major contributor to the pathology of Alzheimer’s disease [30], a chronic progressive neurodegenerative disease with a late onset deemed to be the most common cause of dementia. Alzheimer’s disease has been shown to be followed by epigenetic rDNA silencing, which reduces the number of active rRNA genes, although the total number of ribosomal repeats is not decreased. On the molecular level, rDNA silencing is underpinned by multiple CpG methylation of the rDNA promoter, which is a likely cause of ribosomal deficiency in affected brains. By reducing nucleolar transcription and subsequently decelerating ribosomal turnover, the hypermethylation of the rDNA promoter may participate in the decline of the rRNA component of brain ribosomes, contributing to Alzheimer’s disease-associated synapse loss and resulting in dementia [31]. Cytogenetically, the process of rDNA silencing results in repressive chromatin conformation, reduction in nucleolar volume [32] and the diminished silver-staining of nucleolus organizer regions. These findings are indicative of a global reduction in the activity of ribosomal genes during Alzheimer’s disease [33].

Corroborating the findings obtained on patients with Alzheimer’s disease, mild cognitive impairments (MCI) defined as a transition state from the normal aging process of the brain to dementia and Alzheimer’s disease [34,35] appeared to be linked to low ribosomal gene counts in elderly genomes. A ribosomal deficit was documented in multiple cortical areas of MCI and Alzheimer’s disease subjects [30]. In our recent study, leukocyte rDNA copy numbers were determined in 93 elderly subjects (61–91 years old) with the signs of MCI and 365 healthy controls (16–91 years old) using non-radioactive quantitative hybridization with biotinylated DNA probes. In the group of MCI cases, the rDNA copy number (mean 329 ± 60; median 314 copies) was significantly reduced (*p* < 10^−15^) compared with controls of the same age with preserved cognitive functions (mean 412 ± 79; median 401 copies, N = 168) and younger (16–60 years) controls (mean 426 ± 109; median 416 copies, N = 197) [36].

In summary, those subjects who harbored the middle, adaptive amounts of active genes for rRNA demonstrated better fertility, stress resistance, brain functions and longevity. These observations relate to both subjects with normal karyotypes and carriers of chromosomal abnormalities with increased child mortality and shorter lifetime expectance. There have been no cases so far detected where a negative sign or condition could be linked to a medium rDNA copy number. The medium rDNA abundance is always optimal and adaptive. On the contrary, deviant (either low or high) numbers of ribosomal repeats have been found to belong to risk groups for various kinds of pathologies. Rheumatoid arthritis patients, women with multiple IVF failure, patients with complicated purulent infections and mild cognitive impairment cases show low copy numbers of genes for rRNA or their depressed transcription, whereas schizophrenia cases have been found to harbor a high amount of ribosomal genes in their genomes.

Low rDNA copy numbers in the genome seem to be associated with insufficiently effective ribosome biogenesis. Pregnancy requires intense protein synthesis. In case of a small number of rDNA repeats in the genome, the possibility of the necessary intensification of protein synthesis is limited due to low ribosome content because the rate of rRNA biosynthesis is the major limiting factor of ribosome biogenesis [37], and the contents of rRNA and total proteins are known to be highly correlated. This is why carriers of reduced rDNA copy numbers demonstrate slower development. This phenomenon has been observed in different species. In a classic instance, Drosophila females, homozygous for the *bobbed* mutation, carry a half dose of ribosomal genes, with 130 copies instead of 260 in wild-type flies. The mutant phenotype is characterized by shortened thoracal bristles, the retarded development of larvae and declined fertility and viability resulting in the rapid elimination of mutants from mixed populations with wild-type flies [38,39,40]. Another example is chicken lines with normal, reduced or extended rDNA complements. While a line carrying 66% of the normal complement of ribosomal genes was found to develop and grow normally, the development of embryos of another line containing merely 45% of the normal complement of genes for rRNA in their genomes aborted at early gastrulation [41]. In addition, an investigation of thirteen commercial broiler strains showed that broiler pure lines, which had been directionally selected for faster growth and performance, contained higher rDNA copy numbers than a control line unselected for performance traits [42].

Why is the exaggerated ribosomal repeat count in the genome also disadaptive? The answer is not obvious, as in the case of a low rDNA count. However, this fact is neither surprising nor outstanding. The deleterious and lethal effects of trisomies are well known and are not caused by defects in any gene, but they are exclusively caused by excessive third copies of each normal gene on the triplicated chromosome. Below are some speculations postulating how the excessively high abundance of ribosomal genes can be deleterious.

A large quantity of transcribed rDNA copies in the genome promotes the production of a large number of ribosomes and intense protein synthesis. At the same time, an increase in the number of actively transcribed rDNA copies has been shown to be toxic for the eukaryotic cell. The effect was revealed in experiments on yeast [3]. Interesting findings were obtained from a study on Hutchinson–Gilford progeria syndrome (HGPS), which is caused by a mutation in the gene for lamin A [43]. Due to the mutation, an aberrant form of the protein is synthesized, which has been termed progerin. Upregulated progerin expression in the nucleus is followed by exaggerated ribosome biogenesis [44,45]. The derepressed transcription of rDNA in the cells of HGPS patients is manifested as an increase in the number of mature forms of rRNA. The augmentation of rRNA production in turn results in a substantial increase in the synthesis of ribosomal proteins. Total protein biosynthesis is also upregulated significantly in the cells of HGPS patients [46]. Ribosome biogenesis and translation are two of the most energy-consuming processes that require high amounts of ATP (according to some estimations, up to 80% of the total intracellular ATP) [47]. Some authors believe that protein overproduction induces premature aging in HGPS patients because of a lack of energy, as they are exhausted by this process. The cells of HGPS patients demonstrate very low ATP levels and increased metabolism rates [48,49]. It is obvious that a large amount of transcriptionally active rDNA copies can lead to a similar situation: a lack of energy owing to the hyperactivation of ribosome biogenesis and protein synthesis.

In this context, a study by Tiku et al. (2016) [50] is of interest. The authors showed that an increase in longevity by means of various approaches, including dietary calorie restriction, resulted in a reduction in the expression of both ribosomal RNA and ribosomal proteins. A reduction in ribosome biogenesis intensity was also observed as a result of techniques meant to improve human metabolism. Lowering ribosome biogenesis and the protein synthesis rate can be considered a method of increasing individual longevity.

## 3. The Role of Satellite Repeat Abundance in Modulating Stress, Aging and Pathology

Heat shock is a universal mechanism to restore the homeostatic balance. We have now learned that it can be triggered not only by a thermal impact [51,52,53,54] but also by a diversity of inductors such as irradiation and UV light, azetidine, cadmium salt [55] and hyperosmotic conditions [56], which all launch the same reaction: a transcriptional burst of an array of genes historically called heat shock genes, which encode the specific chaperones [57]. This reaction, known as the heat shock response, is a ubiquitous stress-response mechanism of cell protection against damage. A key vertebrate protein of the heat shock response is the heat shock factor, HSF1 [58,59]. HSF1 is critical for activating heat shock genes by binding to the heat shock elements (HSE) located in the promoters of stress-inducible genes for the chaperones and other protective genes [60].

The other component of the stress response is the temporal silencing of a wide variety of non-HSE-controlled genes. The biological meaning lies in the fact that the cell saves resources in a difficult situation by interrupting most processes and protecting the essential molecules necessary for survival. However, the underpinned mechanics were elusive for a long time. Studies of this side of the adaptive response have resulted in the revision of the “junk DNA” concept. We can now consider it proven that the underlying mechanism requires the transcription of non-coding RNAs from satellite III repeats, previously deemed “constitutive heterochromatin”. The chain of relevant events is now generally outlined, and HSF1 appears to be a central player here as well.

A stress cascade involves the rapid and reversible relocation of HSF1 and some other proteins, particularly splicing factors [13], into so-called nuclear stress granules. They appear, first of all, on the 9q12 locus and chromosome Y [61] and, secondarily, on some other chromosomes carrying SatII/III sequences. Within these structures, HSF1 binds to SatII/III repeats at several pericentromeric regions [62] and launches the transcription of satellite sequences by adding polymerase II to stable transcripts. The stress-induced satellite transcription recruits polymerase II, the histone acetyltransferase CREBBP (also termed CBP) and some splicing factors. As a result, special structures termed “nuclear stress bodies” form. The nuclear stress bodies serve as “traps”, catching the molecules required for gene expression. The resultant sequestration of these molecules provokes the temporary fading of the global genome expression, excluding several stress-inducible genes for the chaperones and other protective genes. Notably, this effect is obviously quantitative; i.e., the more satellite repeats, the stronger the global expression slowdown is.

Unlike ribosomal repeats, SatII/III count can change under stress conditions—favored by the open chromatin conformation necessary for transcription—in the cavalcade of successive cell generations. An amazing phenomenon of SatII repeat expansion was recently found in tumors. It was shown that, after launching the stress-induced transcription of SatII RNA, the resultant repeated transcripts were reverse-transcribed into cDNA intermediates in the form of DNA/RNA hybrids. Then, these SatII RNA-derived DNA (rdDNA) molecules were stably incorporated within pericentromeric loci, leading to SatII copy gain. When the reverse transcriptase activity was inhibited, the SatII copy gain was reduced. Whole-genome sequencing data have revealed that human SatII copy gain is a common phenomenon in primary human colon and kidney tumors [63]. Indirect evidence for somatic satellite expansion during tumor growth was obtained in comparative genomic hybridization (CGH) studies on 75 cases of hepatocellular carcinoma (HCG) in China [64], another 36 histologically confirmed Chinese HCG cases [65] and 33 gastric cancer patients in Saudi Arabia [66], as well as studies on lymphoma and myeloma [67] and HCG related to the hepatitis B virus [68]. Each of them yielded cytologic evidence for the overrepresentation of 1q region (1q12 locus) constituted by satellite repeats, thus suggesting SatIII expansion in cancers.

For SatIII in healthy subjects, similar evidence of copy gain was found in natural aging. In a study of a group of 557 individuals aged 2 to 91 years, the following SatIII content data were obtained in different age groups (mean ± SD, pg/ng): Children, 2–12 years old—14.7 ± 2.7; Adult 1, 17–36 y.o.—21.2 ± 7.2; Adult 2, 37–56 y.o.—21.7 ± 6.0; Adult 3, 57–76 y.o.—22.2 ± 6.0; Senile, 77–91 y.o.—23.5 ± 8.5. One can see a statistically significant continuous trend of growth, which the authors attributed to a satellite copy gain process provoked by inevitable habitual stresses over the course of a lifetime [69]. A similar trend was registered in subjects who had chronic occupational exposure to low-dose ionizing radiation. In unexposed controls, the mean SatIII content was 21 ± 5 pg/ng; in the gamma neutron-irradiated cohort, the mean SatIII content increased up to 24 ± 6 pg/ng; and in the strongly stressed tritium-exposed group, the mean SatIII content achieved 29 ± 26 pg/ng [70].

Thus, tumorogenesis, radiation exposure and aging can be presumably deemed to be triggers for the same copy-gain process. Aging, senescence [71] and cancerogenesis are well-known models of permanent stress. Malignant cells are chronically exposed to various impacts, such as dysregulated metabolism, hypoxia, immune responses, shortages of nutrients and genomic instability [72,73]. An aberrant overexpression of satellite repeats was detected in colon, cervix, pancreatic and other epithelial cancers [74,75].

However, satellite expansion is not a unidirectional and endless process under stressful conditions. Stress-induced SatIII copy number changes in opposite directions were observed in five human skin fibroblast lines, which were exposed to potassium Cr(VI) salt, inducing oxidative stress with a strength quantitatively depending on the Cr(VI) content. Moderate stress at 4 µM of Cr(VI) induced SatIII copy gain in early passages in two lines with a low baseline copy number. Stronger stress at 6 µM reduced the observed effect in these lines. The three other strains with high-baseline SatIII content showed decreased SatIII abundance after exposure to Cr(VI). Thus, the exposed fibroblast lines, which had low initial SatIII content, showed copy augmentation, while the lines with initially high SatIII content otherwise demonstrated a loss of SatIII copies after a stress impact [69]. These data suggested that the stress-induced satellite expansion is limited because a reverse process exists, which results in copy number reduction when the copies become too great.

In line with this hypothesis, a significantly small average number of SatIII copies were found in a large (n = 840) sample of patients with schizophrenia compared with mentally healthy controls (*p* << 10^−17^) [76]. In schizophrenia, elevated reactive oxygen species levels and declined antioxidant statuses in the brain and peripheral tissues have been reported [77,78,79,80,81,82,83,84]. Presumably, the permanent stress in those patients led to copy loss after a hypothetical initial expansion in the pre-clinical period.

Interestingly, fetal hypoxia can be a traumatic factor in some cases of schizophrenia progression in adulthood. They are cases of “organic” etiology, not associated with a strong oxidative or another stress in adulthood. When patients who experienced fetal hypoxia were isolated from the large common sample of schizophrenia patients, this sub-group appeared to contain the same amounts of SatIII DNA in their genomes as healthy controls (*p* > 0.2) [76]. The idea of two reverse processes of satellite copy gain/reduction under stress conditions was embodied in a model called the “pendulum model” [85].

The necessity of restricting the copy numbers of satellite repeats transcribed under stress can be reasonably explained in the following way. After the stress reaction has radically changed the transcriptional landscape on a whole-genome scale, most genes (except for the heat shock genes) undergo global silencing and aberrant splicing, resulting in the suspension of many housekeeping and specialized processes. The biological sense is the resource economy, protection of the chromosomes against damage and reducing the vulnerability of the cellular machinery. All this is surely useful during the stress period but deleterious if it lasts for a long enough time because the human cell is not adapted for long anabiosis. Such a shutdown is just the lesser of two evils.

Even in unstressed cells, low levels of SatIII transcripts have been detected. A normal baseline is approximately 10,000 times lower than after stress induction caused by oxidative stress, heat shock, heavy metals or a hyper-osmotic or ultraviolet impact [56]. When the SatII/III tandem array is too long, the baseline can become substantially elevated, resulting in the constant partial depletion of polII, CREBBP and other factors even without any stress, thus slightly but chronically affecting the crucial cellular functions. This idea was directly proven in experiments on HeLa cells carried out by Goenka and colleagues. First, the authors provoked a knockdown of normal SatIII expression using transient transfection with antisense oligos and, predictably, revealed that the knockdown considerably reduced (by ~70%) the survivability of HeLa cells after a thermal impact compared to cells that had been transiently transfected with control oligos and also exposed to heat shock. After that, they tested a mammalian construct (pcDNA-SatIII), providing constitutive SatIII transcription in normal (stress-free) conditions. The expression of the SatIII construct resulted in a paradoxically lower survival rate compared with HeLa cells transfected with an empty vector. Strikingly, cells that constitutively expressed the cloned SatIII repeats also showed significantly lower survival rates after they were exposed to thermal shock, suggesting the toxic effect of the SatII/III transcripts when expressed ectopically [86].

The activation of heterochromatic satellite transcription is often associated with the derepression of LINE1 transposable elements, which can lead to genomic rearrangements [87]. In human tumors, increased LINE-1 expression is also associated with p53 loss [88]. The loss of the tumor suppressor protein p53 in mouse tumors has been suggested to derepress satellite DNA and SINE (short interspersed nuclear element) transcription, which then triggers a suicidal interferon response [89].

Finally, cytogenetic studies on human 1q12 loci repositioning in normal conditions and during disease have directly demonstrated the abnormal vulnerability of excessively long blocks of satellite repeats due to spatial factors (reviewed in [85]).

## 4. Psychoemotional (Affective) Stress-Induced Changes in the Abundance of SatIII (1q12) and Ribosomal Repeats

A pilot study of affective stress experienced by students during their exams may be an instance of a combined assay of rDNA and SatIII copy numbers, reflecting stress dynamics. Blood samples were taken in a group of n = 42 second-year medical students in stressful conditions (exams) and in a non-stressful control period (vacations). Oxidative stress markers, such as NOX4, 8-oxoG and phosphorylated serine 139 histone H2AX (γ-H2AX), were also monitored and showed affective stress-induced oxidative stress in the participants, but the details are beyond the scope of this review. Psychoemotional stress is a transient stress caused in young and healthy students by a hard educational burden and agitation. The rDNA copy number variability was less than 10% and comparable to the relative standard error of the experiment. Thus, the rDNA content in the genomes of the subjects was a stable genetic trait that did not depend on psychoemotional stress. In contrast to rDNA, the leucocyte content of SatIII significantly changed under stress, demonstrating its maximum 30 days before the exam, when the SatIII abundance was significantly higher in study subjects than in controls, whereas on the day of the exam, and especially after the vacations, the SatIII content was significantly lower in most students compared with the content determined 30 days before the exam and lower than in the controls. Hence, affective exam stress triggered a universal mechanism of oxidative stress, provoking a splash of SatIII copy number dynamics, presumably due to the repeat expansion, while the ribosomal repeat content remained stable. After the release of the stress and the restoration period during the post-examinational vacations, the SatIII content normalized, presumably being reduced by a hypothetical reverse mechanism that counterbalances the satellite repeat expansion [90]. These findings became a validation of the “pendulum model” published earlier [85].

## 5. Concluding Remarks

Above, we reviewed a bulk of facts convincingly showing that the amount of moderate repeats—ribosomal DNA and human satellite III (1q12 loci)—is a factor linked to individual stress resistance, longevity, fertility and the progression of some diseases. A combined assay of the abundance of these two kinds of moderate repeats in the human genome—ribosomal and satellite—can be a useful source of information necessary for personalized prognostics and treatment.

Both repeats show the same tendency, in that the moderate copy number reflects a better situation and prognosis, but the meaning of the relevant CNV is different. rDNA abundance is a marker predetermining the high or low effectiveness of the individual stress response, thereby modulating the individual potential stress resistance, expected lifetime, fertility and predisposition to pathology. This is a constant and inherited trait of the person’s genome, which shows the individual potency and viability for the rest of their lifetime. The closer a personal rDNA count is to the population mean, the better.

Unlike rDNA, the number of satellite repeats is a dynamically changed feature indicating the current state of stress, its duration and acute or chronic character. The moderate abundance means an unstressed situation, while elevated numbers of SatIII copies display a current (acute) stress, and reduced numbers indicate a recently experienced or constant (chronic) stress.

To summarize, rDNA abundance reflects the inherited adaptive reserve in a certain person, whereas the SatIII copy number is an indicator of current, recent or chronic stress. Notably, both indicators are quantitative. Of course, this is a distant prospect for the practical usage of these parameters in personalized medicine, and further studies are warranted. Future studies should be conducted on large samples and designed to calculate correlations, regression and other kinds of quantitative dependence.

## Data Availability

Not relevant to a literature review.

## References

[1] Iskow R.C., Gokcumen O., Lee C. (2012). Exploring the role of copy number variants in human adaptation. Trends Genet..

[2] Kobayashi T. (2011). How does genome instability affect lifespan?. Genes Cells.

[3] Ide S., Miyazaki T., Maki H., Kobayashi T. (2010). Abundance of ribosomal RNA gene copies maintains genome integrity. Science.

[4] Symonová R. (2019). Integrative rDNAomics-Importance of the Oldest Repetitive Fraction of the Eukaryote Genome. Genes.

[5] Moss T., Stefanovsky V.Y. (2002). At the center of eukaryotic life. Cell.

[6] Hannan R.D., Rothblum L.I. (1995). Regulation of ribosomal DNA transcription during neonatal cardiomyocyte hypertrophy. Cardiovasc. Res..

[7] Russell J., Zomerdijk J.C. (2006). The RNA polymerase I transcription machinery. Biochem. Soc. Symp..

[8] Hein A.M., O’Banion M.K. (2012). Neuroinflammation and cognitive dysfunction in chronic disease and aging. J. Neuroimmune Pharmacol..

[9] Hannan K.M., Sanij E., Rothblum L.I., Hannan R.D., Pearson R.B. (2013). Dysregulation of RNA polymerase I transcription during disease. Biochim. Biophys. Acta.

[10] Grummt I., Ladurner A.G. (2008). A metabolic throttle regulates the epigenetic state of rDNA. Cell.

[11] Vourc’h C., Dufour S., Timcheva K., Seigneurin-Berny D., Verdel A. (2022). HSF1-Activated Non-Coding Stress Response: Satellite lncRNAs and Beyond, an Emerging Story with a Complex Scenario. Genes.

[12] Jolly C., Metz A., Govin J., Vigneron M., Turner B.M., Khochbin S., Vourc’H C. (2003). Stress-induced transcription of satellite III repeats. J. Cell Biol..

[13] Metz A., Soret J., Vourc’H C., Tazi J., Jolly C. (2004). A key role for stress-induced satellite III transcripts in the relocalization of splicing factors into nuclear stress granules. J. Cell Sci..

[14] Fritah S., Col E., Boyault C., Govin J., Sadoul K., Chiocca S., Christians E., Khochbin S., Jolly C., Vourc’H C. (2009). Heat-Shock Factor 1 Controls Genome-wide Acetylation in Heat-shocked Cells. Mol. Biol. Cell.

[15] Liapunova N.A., Egolina N.A., Tsvetkova T.G., Veĭko N.N., Kravets-Mandron I.A., Gromova E.V., Kosiakova N.V., Viktorov V.V., Malinovskaia T.N. (2000). Ribosomal genes in the human genome: Contribution to genetic individuality and phenotypic manifestation of gene dosage. Vestn. Ross. Akad. Meditsinskikh Nauk..

[16] Porokhovnik L.N., Lyapunova N.A. (2019). Dosage effects of human ribosomal genes (rDNA) in health and disease. Chromosome Res..

[17] Malinovskaya E.M., Ershova E.S., Golimbet V.E., Porokhovnik L.N., Lyapunova N.A., Kutsev S.I., Veiko N.N., Kostyuk S.V. (2018). Copy Number of Human Ribosomal Genes With Aging: Unchanged Mean, but Narrowed Range and Decreased Variance in Elderly Group. Front. Genet..

[18] Hallgren J., Pietrzak M., Rempala G., Nelson P.T., Hetman M. (2014). Neurodegeneration-associated instability of ribosomal DNA. Biochim. Biophys. Acta.

[19] Porokhovnik L.N., Passekov V.P., Gorbachevskaya N.L., Sorokin A.B., Veiko N.N., Lyapunova N.A. (2015). Active ribosomal genes, translational homeostasis and oxidative stress in the pathogenesis of schizophrenia and autism. Psychiatr. Genet..

[20] Lyapunova N.A., Porokhovnik L.N., Kosyakova N.V., Mandron I.A., Tsvetkova T.G. (2017). Effects of the copy number of ribosomal genes (genes for rRNA) on viability of subjects with chromosomal abnormalities. Gene.

[21] Porokhovnik L.N., Veiko N.N., Ershova E.S., Poletkina A.A., Shmarina G.V., Dolgikh O.A., Klimenko P.A., Klimenko M.P., Avetisova K.G., Kostyuk E.V. (2019). Copy number of ribosomal genes in woman’s genome is associated with IVF outcome and pregnancy complications. Med. Genet..

[22] Mandron I.A., Suchilina M.A., Tsvetkova T.G., Kosyakova N.V. Genomic dosage of active ribosomal genes and severity of dentogenous phlegmons. Proceedings of the VI Congress of Russian Society of Medical Geneticists.

[23] Chestkov I.V., Jestkova E.M., Ershova E.S., Golimbet V.E., Lezheiko T.V., Kolesina N.Y., Porokhovnik L.N., Lyapunova N.A., Izhevskaya V.L., Kutsev S.I. (2018). Abundance of ribosomal RNA gene copies in the genomes of schizophrenia patients. Schizophr. Res..

[24] Oken R.J., Schulzer M. (1999). At issue: Schizophrenia and rheumatoid arthritis: The negative association revisited. Schizophr. Bull..

[25] Eaton W.W., Hayward C., Ram R. (1992). Schizophrenia and rheumatoid arthritis: A review. Schizophr. Res..

[26] Eaton W.W., Byrne M., Ewald H., Mors O., Chen C.Y., Agerbo E., Mortensen P.B. (2006). Association of schizophrenia and autoimmune diseases: Linkage of Danish national registers. Am. J. Psychiatry.

[27] Gorwood P., Pouchot J., Vinceneux P., Puéchal X., Flipo R.M., de Bandt M., Adès J. (2004). Club Rhumatisme et Inflammation. Rheumatoid arthritis and schizophrenia: A negative association at a dimensional level. Schizophr. Res..

[28] Shubaeva N.O. (2004). Molecular Genetic Characteristics of Ribosomal Genes and Cell Death Rates in Patient with Rheumatoid Arthritis. Ph.D. Thesis.

[29] Veiko N.N., Shubaeva N.O., Tsvetkova T.G., Mandron I.A. (2005). The peculiarities of quantitative characteristics of the ribosomal gene complex in patient with severe forms of rheumatoid arthritis. Med. Genet..

[30] Ding Q., Markesbery W.R., Chen Q., Li F., Keller J.N. (2005). Ribosome dysfunction is an early event in Alzheimer’s disease. J. Neurosci..

[31] Pietrzak M., Rempala G., Nelson P.T., Zheng J.J., Hetman M. (2011). Epigenetic silencing of nucleolar rRNA genes in Alzheimer’s disease. PLoS ONE.

[32] Iacono D., O’Brien R., Resnick S.M., Zonderman A.B., Pletnikova O., Rudow G., An Y., West M.J., Crain B., Troncoso J.C. (2008). Neuronal hypertrophy in asymptomatic Alzheimer disease. J. Neuropathol. Exp. Neurol..

[33] Payão S.L., Smith M., Kormann-Bortolotto M.H., Toniolo J. (1994). Investigation of the nucleolar organizer regions in Alzheimer’s disease. Gerontology.

[34] Sanford A.M. (2017). Mild Cognitive Impairment. Clin. Geriatr. Med..

[35] Jongsiriyanyong S., Limpawattana P. (2018). Mild Cognitive Impairment in Clinical Practice: A Review Article. Am. J. Alzheimer’s Dis. Other Dement..

[36] Veiko N.N., Ershova E., Veiko R.V., Umriukhin P.E., Kurmyshev M.V., Kostyuk G.P., Kutsev S.I., Kostyuk S.V. Mild cognitive impairment is associated with low copy number of ribosomal genes in the genomes of elderly people. Front. Genet..

[37] Larson D.E., Zahradka P., Sells B.H. (1991). Control points in eukaryotic ribosome biogenesis. Biochem. Cell Biol..

[38] Ritossa F.M., Atwood K.C., Lindsley D.L., Spiegelman S. (1966). On the chromosomal distribution of DNA complementary to ribosomal and soluble RNA. Natl. Cancer Inst. Monogr..

[39] Ritossa F.M. (1968). Unstable redundancy of genes for ribosomal RNA. Proc. Natl. Acad. Sci. USA.

[40] Mohan J., Ritossa F.M. (1970). Regulation of ribosomal RNA synthesis and its bearing on the bobbed phenotype in *Drosophila melanogaster*. Dev. Biol..

[41] Delany M.E., Muscarella D.E., Bloom S.E. (1994). Effects of rRNA gene copy number and nucleolar variation on early development: Inhibition of gastrulation in rDNA deficient chick embryos. J. Hered..

[42] Su M.H., Delany M.E. (1998). Ribosomal RNA gene copy number and nucleolar-size polymorphisms within and among chicken lines selected for enhanced growth. Poult. Sci..

[43] Eriksson M., Brown W.T., Gordon L.B., Glynn M.W., Singer J., Scott L., Erdos M.R., Robbins C.M., Moses T.Y., Berglund P. (2003). Recurrent de novo point mutations in lamin A cause Hutchinson-Gilford progeria syndrome. Nature.

[44] Goldman R.D., Shumaker D.K., Erdos M.R., Eriksson M., Goldman A.E., Gordon L.B., Gruenbaum Y., Khuon S., Mendez M., Varga R. (2004). Accumulation of mutant lamin A causes progressive changes in nuclear architecture in Hutchinson-Gilford progeria syndrome. Proc. Natl. Acad. Sci. USA.

[45] Shumaker D.K., Dechat T., Kohlmaier A., Adam S.A., Bozovsky M.R., Erdos M.R., Eriksson M., Goldman A.E., Khuon S., Collins F.S. (2006). Mutant nuclear lamin A leads to progressive alterations of epigenetic control in premature aging. Proc. Natl. Acad. Sci. USA.

[46] Buchwalter A., Hetzer M.W. (2017). Nucleolar expansion and elevated protein translation in premature aging. Nat. Commun..

[47] MacInnes A. (2016). The role of the ribosome in the regulation of longevity and lifespan extension. Wiley Interdiscip. Rev. RNA.

[48] Abdenur J., Brown W.T., Friedman S., Smith M., Lifshitz F. (1997). Response to nutritional and growth hormone treatment in progeria. Metabolism.

[49] Merideth M.A., Gordon L.B., Clauss S., Sachdev V., Smith A.C., Perry M.B., Brewer C.C., Zalewski C., Kim H.J., Solomon B. (2008). Phenotype and course of Hutchinson-Gilford progeria syndrome. N. Engl. J. Med..

[50] Tiku V., Jain C., Raz Y., Nakamura S., Heestand B., Liu W., Späth M., Suchiman H.E.D., Müller R.U., Slagboom P.E. (2016). Small nucleoli are a cellular hallmark of longevity. Nat. Commun..

[51] Courgeon A.M., Maisonhaute C., Best-Belpomme M. (1984). Heat shock proteins are induced by cadmium in Drosophila cells. Exp. Cell Res..

[52] Heikkila J.J., Schultz G.A., Iatrou K., Gedamu L. (1982). Expression of a set of fish genes following heat or metal ion exposure. J. Biol. Chem..

[53] Michel G.P., Starka J. (1986). Effect of ethanol and heat stresses on the protein pattern of Zymomonas mobilis. J. Bacteriol..

[54] Yura T., Tobe T., Ito K., Osawa T. (1984). Heat shock regulatory gene (htpR) of Escherichia coli is required for growth at high temperature but is dispensable at low temperature. Proc. Natl. Acad. Sci. USA.

[55] Sengupta S., Parihar R., Ganesh S. (2009). Satellite III non-coding RNAs show distinct and stress-specific patterns of induction. Biochem. Biophys. Res. Commun..

[56] Valgardsdottir R., Chiodi I., Giordano M., Rossi A., Bazzini S., Ghigna C., Riva S., Biamonti G. (2007). Transcription of Satellite III non-coding RNAs is a general stress response in human cells. Nucleic Acids Res..

[57] Åkerfelt M., Morimoto R.I., Sistonen L. (2010). Heat shock factors: Integrators of cell stress, development and lifespan. Nat. Rev. Mol. Cell Biol..

[58] Morimoto R. (1993). Cells in stress: Transcriptional activation of heat shock genes. Science.

[59] Mathew A., Mathur S.K., Jolly C., Fox S.G., Kim S., Morimoto R.I. (2001). Stress-Specific Activation and Repression of Heat Shock Factors 1 and 2. Mol. Cell. Biol..

[60] Cotto J.J., Morimoto R.I. (1999). Stress-induced activation of the heat-shock response: Cell and molecular biology of heat-shock factors. Biochem. Soc. Symp..

[61] Penin J., Dufour S., Faure V., Fritah S., Seigneurin-Berny D., Col E., Verdel A., Vourc’H C. (2021). Chromosome Y pericentric heterochromatin is a primary target of HSF1 in male cells. Chromosoma.

[62] Eymery A., Souchier C., Vourc’H C., Jolly C. (2010). Heat shock factor 1 binds to and transcribes satellite II and III sequences at several pericentromeric regions in heat-shocked cells. Exp. Cell Res..

[63] Bersani F., Lee E., Kharchenko P.V., Xu A.W., Liu M., Xega K., MacKenzie O.C., Brannigan B.W., Wittner B.S., Jung H. (2015). Pericentromeric satellite repeat expansions through RNA-derived DNA intermediates in cancer. Proc. Natl. Acad. Sci. USA.

[64] Peng G., Chai H., Ji W., Lu Y., Wu S., Zhao H., Li P., Hu Q. (2021). Correlating genomic copy number alterations with clinicopathologic findings in 75 cases of hepatocellular carcinoma. BMC Med. Genom..

[65] Wong N., Lam W.-C., Lai P.B.-S., Pang E., Lau W.-Y., Johnson P.J. (2001). Hypomethylation of Chromosome 1 Heterochromatin DNA Correlates with q-Arm Copy Gain in Human Hepatocellular Carcinoma. Am. J. Pathol..

[66] Bibi F., Ali I., Naseer M.I., Mohamoud H.S.A., Yasir M., Alvi S.A., Jiman-Fatani A.A., Sawan A., Azhar E. (2018). Detection of genetic alterations in gastric cancer patients from Saudi Arabia using comparative genomic hybridization (CGH). PLoS ONE.

[67] Le Baccon P., Leroux D., Dascalescu C., Duley S., Marais D., Esmenjaud E., Sotto J.J., Callanan M. (2001). Novel evidence of a role for chromosome 1 pericentric heterochromatin in the pathogenesis of B-cell lymphoma and multiple myeloma. Genes Chromosom. Cancer.

[68] Sy S.M.-H., Wong N., Lai P.B.-S., To K.-F., Johnson P.J. (2004). Regional over-representations on chromosomes 1q, 3q and 7q in the progression of hepatitis B virus-related hepatocellular carcinoma. Mod. Pathol..

[69] Ershova E.S., Malinovskaya E.M., Konkova M.S., Veiko R.V., Umriukhin P.E., Martynov A.V., Kutsev S.I., Veiko N.N., Kostyuk S.V. (2019). Copy number variation of human satellite III (1q12) with aging. Front. Genet..

[70] Korzeneva I.B., Kostuyk S.V., Ershova E., Skorodumova E.N., Zhuravleva V.F., Pankratova G.V., Volkova I.V., Stepanova E.V., Porokhovnik L.N., Veiko N.N. (2016). Human circulating ribosomal DNA content significantly increases while circulating satellite III (1q12) content decreases under chronic occupational exposure to low-dose gamma- neutron and tritium beta-radiation. Mutat. Res. Mol. Mech. Mutagen..

[71] Wei W., Ji S. (2018). Cellular senescence: Molecular mechanisms and pathogenicity. J. Cell. Physiol..

[72] Zhang B., Fan Y., Cao P., Tan K. (2021). Multifaceted roles of HSF1 in cell death: A state-of-the-art review. Biochim. Biophys. Acta (BBA)-Rev. Cancer.

[73] Surman M., Janik M.E. (2017). Stress and its molecular consequences in cancer progression. Postep. Hig. Med. Dosw..

[74] Eymery A., Callanan M., Vourc’h C. (2009). The secret message of heterochromatin: New insights into the mechanisms and function of centromeric and pericentric repeat sequence transcription. Int. J. Dev. Biol..

[75] Ting D., Lipson D., Paul S., Brannigan B.W., Akhavanfard S., Coffman E.J., Contino G., Deshpande V., Iafrate A.J., Letovsky S. (2011). Aberrant overexpression of satellite repeats in pancreatic and other epithelial cancers. Science.

[76] Ershova E.S., Agafonova O.N., Zakharova N., Bravve L.V., Jestkova E.M., Golimbet V.E., Lezheiko T.V., Morozova A.Y., Martynov A.V., Veiko R.V. (2019). Copy number variation of satellite III (1q12) in patients with schizophrenia. Front. Genet..

[77] Emiliani F.E., Sedlak T.W., Sawa A. (2014). Oxidative stress and schizophrenia: Recent breakthroughs from an old story. Curr. Opin. Psychiatry.

[78] Copoglu U.S., Virit O., Kokacya M.H., Orkmez M., Bulbul F., Erbagci A.B., Semiz M., Alpak G., Unal A., Ari M. (2015). Increased oxidative stress and oxidative DNA damage in non-remission schizophrenia patients. Psychiatry Res..

[79] Smaga I., Niedzielska-Andres E., Gawlik M., Moniczewski A., Krzek J., Przegalinski E., Pera J., Filip M. (2015). Oxidative stress as an etiological factor and a potential treatment target of psychiatric disorders. Part 2. Depression, anxiety, schizophrenia and autism. Pharmacol. Rep..

[80] Hardingham G., Do K.Q. (2016). Linking early-life NMDAR hypofunction and oxidative stress in schizophrenia pathogenesis. Nat. Rev. Neurosci..

[81] Koga M., Serritella A.V., Sawa A., Sedlak T.W. (2016). Implications for reactive oxygen species in schizophrenia pathogenesis. Schizophr. Res..

[82] Barron H., Hafizi S., Andreazza A.C., Mizrahi R. (2017). Neuroinflammation and Oxidative Stress in Psychosis and Psychosis Risk. Int. J. Mol. Sci..

[83] Maas D., Valles A., Martens G. (2017). Oxidative stress, prefrontal cortex hypomyelination and cognitive symptoms in schizophrenia. Transl. Psychiatry.

[84] Patel S., Sharma D., Kalia K., Tiwari V. (2017). Crosstalk between endoplasmic reticulum stress and oxidative stress in schizophrenia: The dawn of new therapeutic approaches. Neurosci. Biobehav. Rev..

[85] Porokhovnik L.N., Veiko N.N., Ershova E.S., Kostyuk S.V. (2021). The role of human satellite III (1q12) copy number variation in the adaptive response during aging, stress, and pathology: A pendulum model. Genes.

[86] Goenka A., Sengupta S., Pandey R., Parihar R., Mohanta G.C., Mukerji M., Ganesh S. (2016). Human satellite-III non-coding RNAs modulate heat shock-induced transcriptional repression. J. Cell Sci..

[87] Rodriguez-Martin B., Alvarez E.G., Baez-Ortega A., Zamora J., Supek F., Demeulemeester J., Santamarina M., Ju Y.S., Temes J., Garcia-Souto D. (2020). Pan-cancer analysis of whole genomes identifies driver rearrangements promoted by LINE-1 retrotransposition. Nat. Genet..

[88] Wylie A., Jones A.E., D’Brot A., Lu W.J., Kurtz P., Moran J.V., Rakheja D., Chen K.S., Hammer R.E., Comerford S.A. (2016). P53 genes function to restrain mobile elements. Genes Dev..

[89] Leonova K.I., Brodsky L., Lipchick B., Pal M., Novototskaya L., Chenchik A.A., Sen G.C., Komarova E.A., Gudkov A.V. (2013). P53 cooperates with DNA methylation and a suicidal interferon response to maintain epigenetic silencing of repeats and noncoding RNAs. Proc. Natl. Acad. Sci. USA.

[90] Umriukhin P.E., Ershova E.S., Filev A.D., Agafonova O.N., Martynov A.V., Zakharova N.V., Veiko R.V., Porokhovnik L.N., Kostyuk G.P., Kutsev S.I. (2022). The psychoemotional stress-induced changes in the abundance of SatIII (1q12) and telomere repeats, but not ribosomal DNA, in human leukocytes. Genes.

